# Species-specific basecallers improve actual accuracy of nanopore sequencing in plants

**DOI:** 10.1186/s13007-022-00971-2

**Published:** 2022-12-14

**Authors:** Scott Ferguson, Todd McLay, Rose L. Andrew, Jeremy J. Bruhl, Benjamin Schwessinger, Justin Borevitz, Ashley Jones

**Affiliations:** 1 Research School of Biology, Australian National University, Canberra, ACT Australia; 2 National Herbarium of Victoria, Royal Botanic Gardens Victoria, South Yarra, Victoria, 3004 Australia; 3 School of Biosciences, The University of Melbourne, Parkville, VIC 3010 Australia; 4 Botany & N.C.W. Beadle Herbarium, School of Environmental and Rural Science, University of New England, Armidale, NSW 2351 Australia

**Keywords:** Oxford Nanopore Technologies, PacBio, Long-read sequencing, Basecaller training, Sequencing accuracy, Asphodelaceae basecaller model, Rutaceae basecaller model

## Abstract

**Background:**

Long-read sequencing platforms offered by Oxford Nanopore Technologies (ONT) allow native DNA containing epigenetic modifications to be directly sequenced, but can be limited by lower per-base accuracies. A key step post-sequencing is basecalling, the process of converting raw electrical signals produced by the sequencing device into nucleotide sequences. This is challenging as current basecallers are primarily based on mixtures of model species for training. Here we utilise both ONT PromethION and higher accuracy PacBio Sequel II HiFi sequencing on two plants, *Phebalium stellatum* and *Xanthorrhoea johnsonii*, to train species-specific basecaller models with the aim of improving per-base accuracy. We investigate sequencing accuracies achieved by ONT basecallers and assess accuracy gains by training single-species and species-specific basecaller models. We also evaluate accuracy gains from ONT’s improved flowcells (R10.4, FLO-PRO112) and sequencing kits (SQK-LSK112). For the truth dataset for both model training and accuracy assessment, we developed highly accurate, contiguous diploid reference genomes with PacBio Sequel II HiFi reads.

**Results:**

Basecalling with ONT Guppy 5 and 6 super-accurate gave almost identical results, attaining read accuracies of 91.96% and 94.15%. Guppy’s plant-specific model gave highly mixed results, attaining read accuracies of 91.47% and 96.18%. Species-specific basecalling models improved read accuracy, attaining 93.24% and 95.16% read accuracies. R10.4 sequencing kits also improve sequencing accuracy, attaining read accuracies of 95.46% (super-accurate) and 96.87% (species-specific).

**Conclusions:**

The use of a single mixed-species basecaller model, such as ONT Guppy super-accurate, may be reducing the accuracy of nanopore sequencing, due to conflicting genome biology within the training dataset and study species. Training of single-species and genome-specific basecaller models improves read accuracy. Studies that aim to do large-scale long-read genotyping would primarily benefit from training their own basecalling models. Such studies could use sequencing accuracy gains and improving bioinformatics tools to improve study outcomes.

**Supplementary Information:**

The online version contains supplementary material available at 10.1186/s13007-022-00971-2.

## Background

Since releasing its first sequencing platform in 2012, Oxford Nanopore Technologies (ONT) has significantly improved nanopore chemistry over six iterations (R6.0, R7.0, R7.3, R9, R9.4, and R10.4). Each new chemistry has improved elements of the speed of sequencing, yield, and accuracy [1]. Oxford Nanopore Technologies sequencing platforms (Flongle, MinION, GridION and PromethION) measure the changes in the electrical ion current that occur as DNA moves through a nanopore contained within the flowcell array, which is stored within fast5 files [2, 3]. Basecallers convert this current signal to base pairs of DNA sequence (stored as fastq files) and have been extended to detect methylated bases [4]. Despite the improvements made to the sequencing chemistry and the accuracy at which individual pores' electrical signal is measured, the per-base accuracy still lags behind alternative sequencing platforms [5]. While advances in per-base accuracy of nanopore sequencing have focused on the function and stability of pores and improved basecalling algorithms, an unexplored avenue for greater sequence accuracy could be in the creation of species-specific base calling models.

The signal captured during nanopore sequencing, often referred to as a squiggle, is difficult to convert to DNA bases and has relied on machine learning, which has enabled iterative advances. Early iterations of ONT basecalling software made use of hidden Markov models (HMM), followed by recurrent neural networks (RNN) before settling on connectionist temporal classification (CTC) algorithms [6]. Each iteration of basecaller has improved the accuracy of basecalled reads, using in house training data, as reported by ONT [7]. However, real-world sequencing projects, while seeing significant improvements to basecalling accuracy, do not achieve the reported ONT accuracy [8, 9].

The leading basecalling models produced by ONT, which are included with the basecalling software Guppy (and built into MinKNOW), are trained on a mixture of both native and amplified DNA (gDNA, PCR amplicons, cDNA), obtained from multiple organisms from all kingdoms of life, and viruses [10]. Using these nucleotide mixtures, a CTC model is iteratively trained until accuracy asymptotes. During basecalling, the CTC algorithm classifies discrete sections of the continuous raw signal as its most probable nucleotide [6]. The accuracy of ONT Guppy’s basecalling model potentially suffers a lack of accuracy due to conflicts within the different genome biologies of the training datasets. Produced alongside the basecalled sequence is a per-base quality score. These Phred encoded quality scores show the confidence that the trained CTC algorithm has in classifying a discrete section of signal (squiggle) as an A, T, C or G [8].

A particular issue to basecaller training is likely the differences in DNA methylation motifs and patterns between lineages, especially between domains [11–13]. These methylation differences will likely cause a loss of accuracy and/or certainty when classifying sequence signals [9]. We can analogise this loss of accuracy and/or certainty to the influence of accents within speech recognition [14, 15] as seen, for example, when a speech recognition algorithm is trained on English speakers with a strong accent. The trained speech recognition model is then used to convert to text the speech of another individual with a different accent. Both speak English, but as the pronouncement of syllables varies greatly between training and usage data, the speech recognition algorithm may not produce the correct output.

Additionally, while the basecalling models included in ONT Guppy are trained on a diverse range of species, it is unlikely for a researcher that their species of interest (and its unique genome biology) formed part of the training dataset. Furthermore, as the species used by ONT to train its models are unknown it is impossible to know how different your genome of interest may be from the training data. Another consideration of training accuracy could include DNA extraction and processing protocols, and their impact on sequenced DNA.

We sought to assess if basecalling could be improved by using a single-species and genome-specific trained basecalling model by training basecalling models using R9.4.1 flowcells for both *Phebalium stellatum* (eudicot, Sapindales, Rutaceae; [16]) and *Xanthorrhoea johnsonii* (monocot, Asparagales, Asphodelaceae; [17]), and R10.4 flowcells for *P. stellatum,* two Australian plants whose lineages diverged ~ 136 million years ago [18]. Additionally, by reciprocally basecalling our two species’ ONT sequencing data (*P. stellatum* was basecalled with *X. johnsonii’s* model and *X. johnsonii* was basecalled with *P. stellatum’s* model), we sought to test whether the mixed nature of ONT’s basecalling models could be affecting basecalling accuracy.

## Results

### Assembly of the truth dataset (de novo genome assemblies)

We first filtered and assembled the PacBio Sequel II HiFi reads which served as our truth dataset for model training and basecaller accuracy analysis. By removing short (1 Kbp) and low-quality (Q < 23) HiFi reads, we observed a minimal ~ 2.8 × loss of coverage and a ~ 172.5 bp loss in N50 while raising the average read quality score by ~ 1.6, Additional file 1: Table S1 (pre-assembly filtering of HiFi reads was likely unnecessary and performed due to our high familiarity with ONT data). These filtered HiFi read libraries were assembled with HiFiAsm, and the resulting assemblies contained separate genomes for haplotype 1 and haplotype 2 (assembly statistics reported in Table 1). HiFiAsm also assembles an unphased genome (a more contiguous haplotype merged genome), but as this genome is not used during basecaller training or accuracy analysis we don’t report on its quality or contiguity. Neither of the two species' genomes assembled into full chromosomes, but rather chromosome fragments or contigs, which is typical for genome assembly projects [19]. Both genome assemblies were in agreement with the reported approximations of genome sizes based on C-value calculations; [20]). *Phebalium stellatum* was our most contiguous assembly, having the higher N50 scores (haplotype 1 = 14.12 Mbp; haplotype 2 = 10.14 Mbp) and the longest contigs. *Xanthorrhoea johnsonii* also assembled into a set of highly contiguous haplotypes with good N50 scores (haplotype 1 = 2.33 Mbp; haplotype 2 = 2.22 Mbp) and very long contigs (Table 1).

**Table 1:** HiFi genome assembly statistics

Haplotype	*X. johnsonii*			*P. stellatum*		
	1	2	1 and 2	1	2	1 and 2
Genome size (Mbp)	1443.23	1436.42	2879.65	654.91	593.36	1248.27
N50 (Mbp)	2.33	2.22	2.25	14.12	10.14	12.51
Contig count	1940	1599	3539	1323	341	1664
Longest contig (Mbp)	21.45	29.62	29.62	39.44	28.58	39.44
Shortest contig (Kbp)	16.31	21.27	16.31	14.57	10.18	10.18

Statistics describing the assembly (both haplotypes and combined haplotypes) contiguity for *Phebalium stellatum* and *Xanthorrhoea johnsonii*

After assembly, both haplotype 1 and haplotype 2 for each genome assembly were joined in a single fasta file (i.e., not collapsed or merged), creating a single pseudo-diploid genome for *P. stellatum* and *X. johnsonii*. These pseudo-diploid genomes became our truth datasets and were used for both basecaller training and model accuracy analysis. To our knowledge, this is the first time diploid reference genomes have been used for training, which is key to separate allele variation among haplotypes from sequencing errors. Without diploid resolution training cannot improve beyond the heterozygosity rate.

### Basecaller training

As basecaller training is limited by available compute resources we began training by subdividing our fast5 sequencing files into smaller datasets. Reads were divided into subsets that would finish basecalling within our maximum allowed job run time. For *P. stellatum* R9.4.1 we created three equally sized read sets containing a total of 1,776,000 reads. Similarly, for *P. stellatum* R10.4 we created three equally sized read sets containing a total of 1,944,674 reads, and for *X. johnsonii* we created two equally sized training with a total of 1,767,914 reads. Subdivision of reads was necessary due to the number of reads obtained for each of the plant species. However, this may not be necessary depending on the number of reads obtained for basecaller training (i.e., from a MinION), or if compute resources aren’t limiting.

Each subset of reads for training were basecalled with ONT Bonito and subsequently, a basecaller model was trained. Basecalling subsets of reads resulted in the total generation of 449 Mbp of sequence for *P. stellatum* R9.4.1, 1,318 Mbp for *P. stellatum* R10.4, and 540 Mbp for *X. johnsonii* R9.4.1. For all datasets, we trained a model on a single subset of reads and iteratively refined this model with all other training read subsets. For all R9.4.1 datasets we used the following parameters: epochs: 15, learning_rate: 0.0002, batch_size: 100, and num_chunks: 0. Training *P. stellatum* R10.4 required a batch_size = 64, as reads were longer, all other parameters were the same as for R9.4.1 training. Complete species-specific basecaller models were exported into a Guppy-compatible format and the configuration file for the bonito model dna_r10.3_450bps_sup was modified appropriately for each model. For details on job run times and memory usage, see Additional file 1: Table S2.

**Fig. 1:**
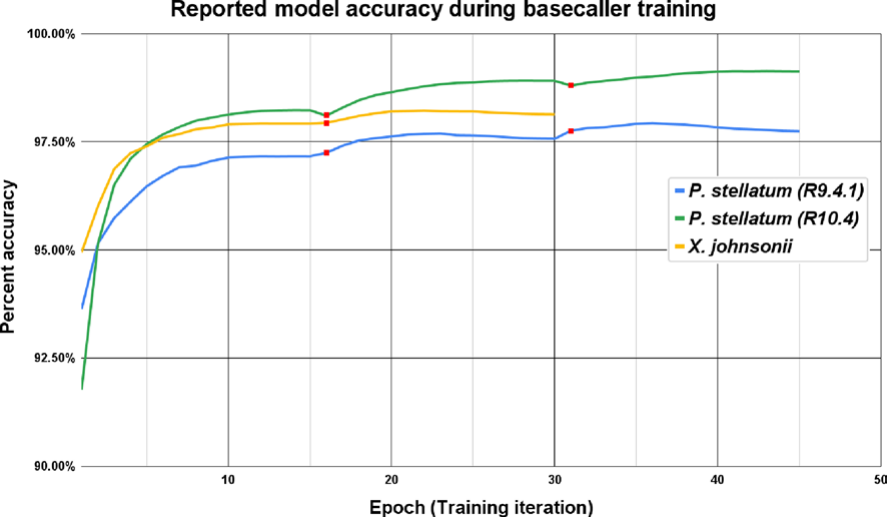
Bonito reported model accuracy during basecaller training. Red dots indicate when training on the next read subset commenced

At the beginning of each epoch (training iteration), ONT Bonito reserves a collection of test reads for model evaluation. These test reads are basecalled after each epoch, then aligned to the truth dataset to assess the model accuracy. Plotting these accuracies demonstrate the models did improve and revealed when asymptotes were approached during training (Fig. 1). For both *P. stellatum* R9.4.1 and *X. johnsonii* models, training peaked after 10 epochs. *Phebalium stellatum* R10.4 training peaked at 15 epochs. All models were improved by training on additional subsets of reads. The models used for the remainder of this study were trained with 15 epochs on each subset of reads.

### R9.4.1 model quality and accuracy

**Fig. 2:**
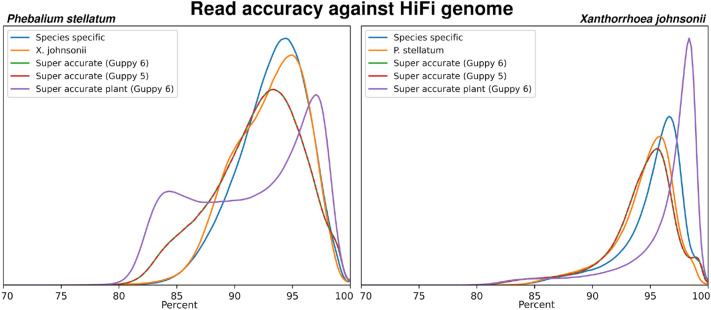
Distribution of R9.4.1 reads accuracies from each basecalled dataset (in percentages). Left figure shows the distribution of all R9.4.1 *Phebalium stellatum* basecalled dataset accuracies compared to the pseudo-haploid HiFi genome (truth set). Right figure shows the distribution of all R9.4.1 *Xanthorrhoea johnsonii* read dataset accuracies compared to the pseudo-haploid HiFi genome (truth set)

**Fig. 3:**
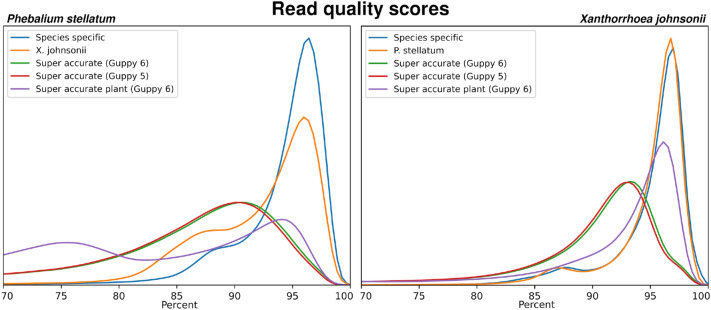
Distribution of R9.4.1 read quality scores, in percentages. Left figure shows the distribution of all R9.4.1 *Phebalium stellatum* read library average read quality scores as reported by guppy. Right figure shows the distribution of all R9.4.1 *Xanthorrhoea johnsonii* read library average read quality scores as reported by guppy

Having trained a species-specific basecaller model for each of the two study plants using our R9.4.1 reads, we next sought to evaluate the improvements in basecalling accuracy. Basecalling of fast5 sequences was performed with multiple versions and models of ONT Guppy for each plant; version: 5.0.7 using the super-accurate model, version: 6.0.2 using the super-accurate model, version: 6.1.2 using the only available ONT plant model, and version: 6.0.2 and using both of our species-specific plant models. This included basecalling each plant with the relevant species-specific model but also the model for the other plant, i.e., *P. stellatum* reads were basecalled with the *X. johnsonii* model and *X. johnsonii* reads were basecalled with the *P. stellatum* model. These basecalled datasets will be referred to as Guppy-5, Guppy-6, Guppy-6-plant, *P. stellatum* and *X. johnsonii,* respectively. For all basecalled datasets, we calculated the average quality score per-read and average read identity compared to the truth dataset (diploid HiFiAsm genome), which are presented in Table 2 and Fig. 2. Each basecalled dataset contained all reads, no filtering was performed. The distributions of quality scores are presented in Fig. 3. Quality score distributions and statistics are also displayed as Phred scores, in Additional file 1: Fig. S1 and Table S3.

**Table 2:** Summary of quality statistics for the R9.4.1 basecalled datasets, in percentages

Basecaller model	*P. stellatum* (R9.4.1)			*X. johnsonii*		
	Quality score	Read identity	R^2^	Quality score	Read identity	R^2^
Guppy 5 super accurate	86.35% ± 45.92%	91.96% ± 3.83%	0.777	90.16% ± 48.12%	94.15% ± 3.12%	0.801
Guppy 6 super accurate	86.68% ± 46.75%	91.96% ± 3.83%	0.777	90.47% ± 48.75%	94.15% ± 3.12%	0.801
Guppy 6 super accurate—plant	84.73% ± 50.92%	91.47% ± 3.50%	0.850	92.81% ± 54.60%	96.18% ± 3.50%	0.900
Reciprocal species-specific	93.00% ± 50.83%	92.98% ± 3.16%	0.577	95.55% ± 45.02%	94.43% ± 2.69%	0.787
Species-specific	94.83% ± 47.35%	93.24% ± 3.00%	0.648	95.59% ± 48.46%	95.16% ± 2.74%	0.805

Average read quality scores and read identity for all *Phebalium stellatum* (R9.4.1) and *Xanthorrhoea johnsonii* basecalled datasets. Average read quality scores were calculated per-read, and the overall average calculated. Read identity is calculated per-read against the HiFi genome and averaged. Averages are shown with standard deviations. For reciprocal species-specific models, *P. stellatum* reads were basecalled with the *X. johnsonii* model and *X. johnsonii* reads were basecalled with the *P. stellatum* model. R^2^ values show the correlation between average read quality score and read identity, calculated by linear regression

The species-specific models were found to have a notable increase in accuracy compared to both the ONT Guppy-5 and Guppy-6 models, which were nearly identical. Compared to the Guppy-6 model, our species-specific models increased average read quality scores by 8.15% for *P. stellatum* (86.68–94.83%) and 5.12% for *X. johnsonii* (90.47–95.59%). This improvement for *X. johnsonii* resulted in the highest average read quality observed in these datasets, which was 95.59%. These quality score improvements held true when compared to the PacBio HiFi genome reference (truth set), as we also observed increased average read identities of 1.28% for *P. stellatum* and 1.01% for *X. johnsonii*. Interestingly, basecalling *X. johnsonii* with the *P. stellatum* model gave near equal improvements in average read quality score and read identity as basecalling with the species-specific model for *X. johnsonii*. This observation was not symmetrical, as basecalling *P. stellatum* with the *X. johnsonii* model produced results of lesser quality than the species-specific model. Basecalling our plants with the Guppy-6-plant model, the only publicly available plant model, gave mixed results. Firstly, as seen in Fig. 2, a large portion of reads appeared to have the highest read qualities, but also a large portion appeared to have the worst read quality scores, in particular for *P. stellatum*. This model did produce the highest average read identity observed, 96.18% for *X. johnsonii* (compared to 95.59% with the species-specific model), but also the worst read identity observed out of all the models tested, 91.47% for *P. stellatum* (compared to 93.24% for the species-specific model). This may reflect the origin of Guppy-6-plant training data, for instance, Guppy-6-plant may have been trained on a monocot species and not eudicot. However, the Guppy-6-plant model, like our species-specific models, demonstrated the value of further developing plant basecalling models.

Using linear regression we determined the correlation of average read quality score to average read identity, Table 2. None of the basecaller model average read quality scores, including our species-specific models, were found to be highly correlated with average read identity. Guppy-6-plant had the highest correlation (*P. stellatum* R2 = 0.850; *X. johnsonii* R^2^ = 0.900) and when we applied the species-specific models to the other plant (*P. steallum* to *X. johnsonii* and the reverse) the lowest (*P. stellatum* sequencing data R^2^ = 0.577; *X. johnsonii* sequencing data = 0.787). Interestingly, all *X. johnsonii* read datasets were more highly correlated with read identity than each equivalent *P. stellatum* dataset. For regression scatter plots see Additional file 1: Figs. S2 and S3; and for phred scores see Additional file 1: Figs. S4 and S5.

Lastly, we investigated the average read lengths for each basecalled dataset (Table 3 and Additional file 1: Fig. S6). Interestingly, we found that the datasets generated with Guppy-5, Guppy-6 and Guppy-6-plant models had considerably longer reads than our species-specific models (*P. stellatum* N50: ~ 46 Kbp compared to ~ 42 Kbp; *X. johnsonii* N50: ~ 40 Kbp compared to ~ 38 Kbp). A potential explanation for these models having the longest reads but also the lowest quality in many instances, could be due to the error profile of ONT sequencing resulting in the erroneous insertion of indels, and therefore lower quality reads are expected to be longer in length.

**Table 3:** Read lengths of R9.4.1 datasets

Basecaller model	*P. stellatum* (R9.4.1)		*X. johnsonii*	
	Average (Kbp)	N50 (Kbp)	Average (Kbp)	N50 (Kbp)
Guppy 5 super accurate	28.98 ± 24.87	45.89	25.49 ± 21.91	39.57
Guppy 6 super accurate	28.99 ± 24.87	45.89	25.49 ± 21.91	39.57
Guppy 6 super accurate—plant	26.15 ± 24.47	45.39	23.48 ± 21.81	39.52
Reciprocal species-specific	26.02 ± 22.80	41.40	24.71 ± 21.10	38.19
Species-specific	26.76 ± 23.07	42.28	24.81 ± 21.26	38.42

Average and N50 read lengths for each dataset. Averages are shown with standard deviations. Reciprocal species-specific are datasets where *Phebalium stellatums* reads were basecalled with the *X. johnsonii* model and *Xanthorrhoea johnsoniis* reads were basecalled with the P. stellatum model

### R10.4.1 model quality and accuracy

In addition to ONT sequencing with standard SQK-LSK110 ligation kits and R9.4.1 flow cells, we performed sequencing for *P. stellatum* with the newer ONT chemistry marketed as Q20+ sequencing (99%), using SQK-LSK112 ligation kits and R10.4 flow cells (containing E8.1 pores). We trained an additional *P. stellatum* model for this new sequencing dataset and assessed any potential accuracy gains made.

**Fig. 4:**
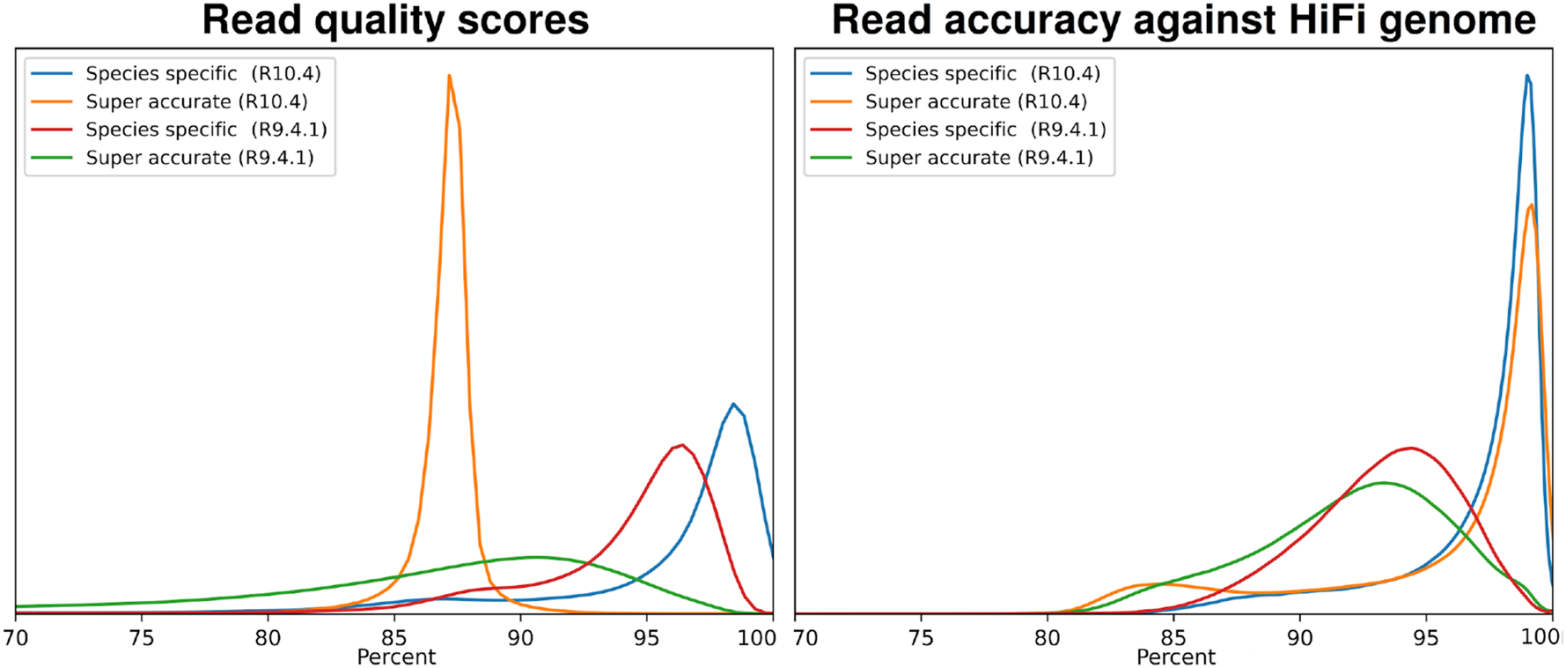
Distribution of R10.4 sequencing quality statistics, in percentages. Left figure shows the distribution of average read quality scores (obtained from basecalled fastq files) for *Phebalium stellatum* basecalled datasets. **B** Distribution of read identities (compared to the PacBio HiFi genome (truth set)) for all *P. stellatum* basecalled datasets

After training the new *P. stellatum* R10.4 species-specific model, we performed basecalling, calculated average read quality scores and average read identity compared to the truth dataset (Fig. 4 and Table 4). Basecalling of R10.4 sequencing data was performed with ONT Guppy version 6.0.6 super-accurate and also basecalled with our trained species-specific *P. stellatum* R10.4 model. These models will be referred to as Guppy-6-R10.4 and *P. stellatum* R10.4, respectively. Same as our previous analyses, we included all basecalled reads in the analysis (no filtering performed), and quality score distributions and statistics are also displayed as Phred scores, in Additional file 1: Fig. S7 and Table S4.

**Table 4:** R10.4 Summary of basecalling quality statistics, in percentages

Basecaller model	Quality score	Read identity	Read N50 (Kbp)	Average read length (Kbp)	R^2^
Guppy-6-R10.4	90.00% ± 4.72%	95.46% ± 5.05%	39.19	22.97 ± 22.39	0.745
Species-specific R10.4	96.73% ± 67.96	96.87% ± 3.26%	37.24	20.19 ± 21.36	0.893

Average read quality scores and read identity for all *Phebalium stellatum* R10.4 read libraries. Average read quality scores were calculated per-read, and the overall average calculated. Read identity is calculated per-read against the HiFi genome and averaged

Our species-specific *P. stellatum* R10.4 model outperformed our previous species-specific model for *P. stellatum* (R9.4.1), increasing average read quality scores by 1.90% and increasing average read identities by 3.63%. Using this model, we achieved our highest accuracy metrics observed in our plants, with an average read quality score of 96.73% and average read identities of 96.87%. We also observed that Guppy-6-R10 outperformed the previous Guppy-6 analyses on both quality metrics, having both a higher average read quality score (+ 3.32%) and higher average read percent identity (+ 3.50%).

Using linear regression we calculated the correlation of average read quality score to average read identity, presented in Table 4 (for regression scatter plots see Additional file 1: Figs. S8 and S9). While the species-specific *P. stellatum* R10.4 model average read quality scores were found to be more correlated with read identity than Guppy-6-R10, neither of the models was highly correlated with average read identity. This suggests that predicted read qualities are not sufficient indications of actual read identities.

Lastly, we performed basecalling of the R10.4 sequencing data with Guppy version 6.0.6 super-accurate duplex, where the first strand template and the second strand complement is collapsed into potentially higher quality reads. After examination of the quality of duplex reads, unfortunately, < 6% of reads were duplex and did not align to the genome. These were not further considered and reflect the early stages of this technology (see Additional file 1: Additional Results).

## Discussion

Oxford Nanopore’s long-read sequencing has become a valuable tool to the research community [21–24], due to the flexible format of the sequencing, length of reads, low cost, and comparable ability to assemble genomes [25]. There have been ongoing improvements to the accuracy of ONT sequencing, particularly for humans and bacterial genomes; however, improvements in plant basecalling have been limited. Here we trained two R9.4.1 plant species-specific basecaller models and one additional for R10.4 flow cells, attempting to raise the accuracy of ONT read libraries and examine any accuracy improvements gained. Additionally, analysis of our basecalling results was performed to investigate if ONT’s practice of releasing a single basecalling model is appropriate, based on an unknown suite of organisms used for model training. The single basecalling model is provided by ONT for use in all species within all kingdoms, potentially confounding the unique genome biology (especially high level of methylation in native plant DNA) of studied organisms and producing basecalled sequence of lower quality than could be achieved with a single-species or lineage specific model [9].

Analysis of both our R9.4.1 and R10.4 species-specific basecalling models demonstrates that basecalling ONT with these models improves read accuracy. Additionally, a comparison of equivalent R9.4.1 to R10.4 sequencing datasets (Guppy super-accurate to Guppy super-accurate) demonstrates that sequencing with version 12 chemistry and R10.4 flow cells are of superior accuracy to those of R9.4.1. The highest read accuracies were achieved by sequencing with the R10.4 kit and basecalling with the species-specific model. Helping to confirm our increased read accuracy, we also observed a slight reduction in average read length, likely resulting from a reduction in indel errors within our reads. Although sequencing with this new chemistry and R10.4 flow cells has a lower output, we believe this is an advantageous pursuit and hypothesise that highly accurate long-reads will be more beneficial to most genomic research applications than higher output of low accuracy reads. For example, large scale pan-genome studies may be able to reduce coverage if using high-accuracy species-specific basecalled R10.4 reads [26].

While both our study species originate within Australia, they are separated by ~ 136 million years of evolution, since the divergence of the monocot and eudicot lineages [18]. Due to the age of the lineage divergence, it is expected that the genomes of these two highly different species would contain different genome biology. Despite these expected genomic differences, when using a species-specific plant model to cross basecall a different plant, the resulting basecalled dataset achieved higher accuracy than that of the Guppy super-accurate model. This demonstrated the importance of developing plant models, which perform better than mixed models primarily based on non plant organisms.

With Guppy version 6.1.2, ONT introduced a plant-specific basecaller model. Examination of this model gave mixed results, *P. stellatum* basecalled reads were of low-quality, while *X. johnsonii* basecalled reads were of high-quality. The different results obtained by our two species are likely due to their evolutionary distances to the plant model training species, and differences between their abundance and diversity of their DNA methylation. Currently, the training species that ONT use are not well documented; however, the plant basecaller model does include maize within its training dataset [27]. The inclusion of maize likely explains *X. johnsonii*’s read accuracy outperforming *P. stellatum* when basecalled with the plant model. *Xanthorrhoea johnsonii*’s unique genome biology more closely resembles maize (both are monocots) than does *P. stellatum*’s*.*

With the introduction of the newer chemistries and flow cells, ONT has enabled the generation of higher accuracy duplex reads. However, analysis of *P. stellatum* duplex reads was not possible, as the majority of reads failed to align to the *P. stellatum* PacBio HiFi genome. The cause of alignment failure is currently unknown. During construction of duplex reads, template and complement strands appeared to have been correctly identified and collapsed. As more data becomes available, efforts to examine duplex reads will continue.

In our study, read quality scores for all the basecalling models tested were found to be a poor indicator of read accuracy. This observation has implications concerning quality assessment and filtering of read libraries with quality scores. Before using read libraries, researchers typically quality screen reads with read per-base quality scores, the only quality metric available. Decisions on read trimming and filtering are made based solely on read quality scores, which, if basecalled using a model trained on mixed data may result in the unnecessary removal of accurate reads (Table 4 and Additional file 1: Table S3). Loss of usable high-quality reads could impact downstream analysis and study feasibility, and will likely increase cost.

The method we used to train our basecaller model used both HiFi (PacBio) and PromethION (ONT) sequencing data. As such a dataset comes at a large cost, many projects may not be able to justify such a study. However, as the truth dataset needs to be highly accurate, contiguous, and haplotype resolved, assembling a HiFi genome currently provides the best truth dataset. The per-base accuracy and haplotype resolution of an ONT assembled genome will not be adequate to use as a truth dataset. Of particular note is the requirement to include both homologous sets of chromosomes within the truth dataset, ensuring that the identity of all reads can be established. To obtain the best results from basecaller training, a HiFi genome should be used as the truth dataset, and ONT reads for basecalling. However, it might not be realistic to spend such an investment in HiFi sequencing to obtain a truth dataset. Our results show that this truth dataset doesn’t need to be from the same species, or even the same lineage, to obtain improved basecalling accuracy. Indeed, for basecalling an angiosperm, a model trained on any flowering plant would likely provide an improvement over the default super-accurate model, though phylogenetically closer species would be preferable. As more both HiFi and ONT sequence data are accumulated for more species, this question may be addressed with further resolution.

## Conclusions

The development of species-specific basecalling models can have a substantial impact in improving the accuracy of long-read sequencing. This improved accuracy has the potential to be beneficial to many research questions and genomic applications. Landscape-scale studies, metabarcoding studies or those examining the genetics of large groups would be the ideal candidate studies to investigate the value add and impact of species-specific basecaller training. Such studies, with improved long-read sequencing accuracies, could make use of new bioinformatics tools to better variant call both point mutations (SNPs) and structural variations (SV) e.g., Longshot [28], medaka [29], NanoCaller [30], and pepper [31]. To date, ONT has focused on a single mixed-species basecalling model that, due to conflicting genome biology, may reduce the results of basecalling. We recommend that long-read providers and genomic researchers investigate the appropriateness of having several lineage-specific basecalling models. For example, a model trained for each domain, kingdom, or phylum or even family and genus. Alternatively, the research community could begin building and sharing species- or lineage-specific basecaller models, as we have done with this study. Basecaller model sharing could be done similarly to how other sequence resources are shared, benefiting the entire genomic community.

## Methods

### DNA extraction and sequencing

#### Tissue collection

Both *Phebalium stellatum* and *Xanthorrhoea johnsonii* are Australian plants growing at the Australian National Botanic Gardens, Canberra, Australia. Living collections accession numbers CANB 914043 (section np1) and CBG 8311086.1 (section 15F) respectively. *Phebalium stellatum* has been vouchered at the N.C.W. Beadle Herbarium, UNE, Armidale, Australia; voucher herbarium catalogue number NE 109286. *Xanthorrhoea johnsonii* has been vouchered at the Australian National Herbarium, Canberra, Australia; voucher herbarium catalogue number CBG 8900857. Leaf tissue was collected and stored at − 80 °C until DNA extraction.

#### High-molecular weight DNA extraction for long-read sequencing

High-molecular weight DNA was extracted with a magnetic bead-based protocol which is described in [32]. In brief, leaf material was ground with a mortar and pestle under liquid nitrogen, homogenate was washed with a sorbitol buffer, an SDS buffer lysis buffer was used followed by protein precipitation with potassium acetate, then DNA was bound to magnetic beads for further washing with ethanol before elution.

##### Long-read native DNA sequencing with Oxford Nanopore Technologies

High-molecular weight DNA was size selected for fragments ≥ 20 kb using a PippinHT (Sage Science). An Oxford Nanopore Technologies native DNA sequencing library was constructed according to the manufacturer's protocol ‘Genomic DNA by Ligation (SQK-LSK110)’. Sequencing was performed on an ONT PromethION using a FLO-PRO002 R9.4.1 flow cell. Additionally, ONT Q20+ sequencing was done with *P. stellatum* using the new ligation kit ‘Genomic DNA by ligation using the Q20+ Kit (SQK-Q20EA)’, now renamed SQK-LSK112, on the PromethION using a FLO-PRO112 R10.4 flow cell. When sequencing declined (low active pore count, approximately 24 h), the flow cell was treated with DNAse I, primed again and more library was loaded, according to the manufacturer’s ‘Flow Cell Wash Kit (EXP-WSH004)’. This was performed at least twice to maximise total sequencing output of the flow cell, until the flow cell was expended.

#### Single molecule, real-time (SMRT) sequencing with Pacific Biosciences

High-molecular weight DNA was sheared to approximately 18 kb fragments with a Megaruptor 3 (Diagenode), using cycle 1 at 31× speed and cycle 2 at 32× speed. The DNA was then subjected to a 0.5× reaction of NEBNext FFPE DNA Repair Mix (New England BioLabs M6630) for 10 min at room temperature and then size selected for fragments ≥ 15 kb with a BluePippin (Sage Science). A PacBio SMRTbell library was prepared according to the manufacturer’s ‘SMRTbell Express Template Prep Kit 2.0′ (Pacific Biosciences). Sequencing was performed on a PacBio Sequel II using an 8 M SMRT cell, with the circular consensus sequencing (CCS) mode to generate high-accuracy HiFi reads.

### Species-specific basecaller model training

Basecaller training requires a truth dataset to train and assess model accuracy. The truth dataset is ideally a highly accurate, haplotype-resolved and complete genome sequenced from the same sample used for training. Using separate parental chromosomes for training allows the sequencing error rate to become lower than the heterozygosity threshold, and is essential for basecaller training of highly heterozygous (2–5%) wild species. While the availability of a high-quality genome for training is not possible for every project or organism, we generated one for each of our study species here to enable benchmarking of the methodology.

Our truth dataset was created by assembling high-accuracy HiFi reads with HiFiAsm (default parameters; version: 0.16.1-r375; [33]). Pre-assembly, we filtered our reads, removing all reads < 1 Kbp in length and < q23 (< 99.5% accurate). HiFiAsm assembles three genomes, haplotype 1, haplotype 2, and unphased [33]. Haplotypes 1 and 2 were placed into a single fasta file, creating a haploid genome for our truth dataset.

Basecaller training began by subdividing our fast5 sequence files into smaller training datasets to suit our computing environment. Training data subdivision may not be needed if sequenced on a MinION. Basecaller training is performed by Bonito (versions: *P. stellatum* R9.4.1: 0.4.0; *X. johnsonii* R9.4.1: 0.4.0; *P. stellatum* R10.4: 0.5.3; [34]), an ONT provided CTC basecaller trainer. Basecaller training began by basecalling fast5 subdivisions with Bonito, using the appropriate basecalled model (dna_r9.4.1 and dna_r10.4_e8.1_hac@v3.4), and the parameters -save-ctc and -reference. Basecalling is performed to identify the true sequence of each read by alignment to the truth dataset (HiFi genome). Next, our species-specific models are trained with Bonito. Parameters for Bonito model training are specific to your computing environment. In particular, batch_size and num_chunks are used to specify the number of training reads and length to split your reads into, respectively. These two parameters specify the amount of data used during training and are limited by GPU memory size. Epochs (learning iterations) and learning_rate (step size) can also be tuned based on compute time restrictions. A smaller learning_rate and more epochs can achieve better results but require more computing time. Bonito’s pretrained parameter was used to train subdivided sequences. Using pretrained, the model trained in the previous training run will become the input for the next run.

As we used Guppy to basecall fast5 files, we exported a Guppy-compatible model of our species-specific basecaller models. In addition to a JSON file, Guppy models also require a config file. Bonito’s dna_r10.3_450bps_sup.cfg file was modified, renamed, and placed within Guppy’s data directory for use.

### Assessment of basecaller model quality

Having basecalled our fast5 files, we next assessed the quality and accuracy of the resulting basecalled reads. Two methods were used to assess the accuracy of our models and determine if they could outperform the Guppy models. First, the internal basecalling quality was assessed by average read quality scores. Using NanoPlot (version 1.1.0; [35]) read statistics were compiled, allowing us to compare read lengths, average quality scores, and total output between different basecaller models. Secondly, we assessed actual read accuracy by comparing reads to the HiFi “truth” diploid reference dataset. Basecalled reads are aligned to our truth dataset with minimap2 (version: 2.22; parameters: -ax map-ont; [36]) and using Promixio [37] read identity (percent similarity) calculated for all reads. We report quality scores as both percent and as Phred scores for comparison on a log scale.

## Supplementary Information

**Additional file 1. **Additional results, additional tables S1–S4 and additional figures S1–S9.

## Data Availability

All sequencing data used in this study are available from Bioplatforms Australia data portal: https://data.bioplatforms.com/organization/about/bpa-plants. Analysis scripts have been deposited within our github repository: https://github.com/fergsc/ONT-Basecaller-training. All trained ONT basecaller model have been deposited in FigShare and are available at: https://figshare.com/projects/Plant_species-specific_basecaller_improves_actual_accuracy_of_nanopore_sequencing/144492.

## References

[1] Wang Y, Zhao Y, Bollas A, Wang Y, Au KF (2021). Nanopore sequencing technology, bioinformatics and applications. Nat Biotechnol.

[2] Fuller CW, Kumar S, Porel M, Chien M, Bibillo A, Stranges PB (2016). Real-time single-molecule electronic DNA sequencing by synthesis using polymer-tagged nucleotides on a nanopore array. Proc Natl Acad Sci.

[3] Silvestre-Ryan J, Holmes I (2021). Pair consensus decoding improves accuracy of neural network basecallers for nanopore sequencing. Genome Biol.

[4] Simpson JT, Workman RE, Zuzarte PC, David M, Dursi LJ, Timp W (2017). Detecting DNA cytosine methylation using nanopore sequencing. Nat Methods.

[5] Amarasinghe SL, Su S, Dong X, Zappia L, Ritchie ME, Gouil Q (2020). Opportunities and challenges in long-read sequencing data analysis. Genome Biol.

[6] Wan YK, Hendra C, Pratanwanich PN, Göke J (2022). Beyond sequencing: machine learning algorithms extract biology hidden in nanopore signal data. Trends Genet.

[7] Rang FJ, Kloosterman WP, de Ridder J (2018). From squiggle to basepair: computational approaches for improving nanopore sequencing read accuracy. Genome Biol.

[8] Delahaye C, Nicolas J (2021). Sequencing DNA with nanopores: troubles and biases. PLoS ONE.

[9] Wick RR, Judd LM, Holt KE (2019). Performance of neural network basecalling tools for Oxford Nanopore sequencing. Genome Biol.

[10] Oxford Nanopore Technologies. How basecalling works. Oxford Nanopore Technologies. http://nanoporetech.com/how-it-works/basecalling. Accessed 27 Mar 2022.

[11] Law JA, Jacobsen SE (2010). Establishing, maintaining and modifying DNA methylation patterns in plants and animals. Nat Rev Genet.

[12] Catania S, Dumesic PA, Pimentel H, Nasif A, Stoddard CI, Burke JE (2020). Evolutionary persistence of DNA methylation for millions of years after ancient loss of a de novo methyltransferase. Cell.

[13] Lewis SH, Ross L, Bain SA, Pahita E, Smith SA, Cordaux R (2020). Widespread conservation and lineage-specific diversification of genome-wide DNA methylation patterns across arthropods. PLOS Genet..

[14] Ghorbani S, Bulut AE, Hansen JHL. Advancing multi-accented LSTM-CTC speech recognition using a domain specific student-teacher learning paradigm. ArXiv180906833 Eess. 2019. http://arxiv.org/abs/1809.06833. Accessed 27 Mar 2022.

[15] Shi X, Yu F, Lu Y, Liang Y, Feng Q, Wang D, et al. The accented english speech recognition challenge 2020: open datasets, tracks, baselines, results and methods. ArXiv210210233 Cs Eess . 2021. http://arxiv.org/abs/2102.10233. Accessed 27 Mar 2022.

[16] Telford IRH, Sadgrove NJ, Bruhl JJ (2018). Three new species segregated from *Phebalium squamulosum* subsp. squamulosum (Rutaceae) based on morphological and phytochemical data. Muelleria..

[17] McLay TGB, Ladiges PY, Doyle SR, Bayly MJ (2021). Phylogeographic patterns of the Australian grass trees (Xanthorrhoea Asphodelaceae) shown using targeted amplicon sequencing. Aust Syst Bot.

[18] Givnish TJ, Zuluaga A, Spalink D, Soto Gomez M, Lam VKY, Saarela JM (2018). Monocot plastid phylogenomics, timeline, net rates of species diversification, the power of multi-gene analyses, and a functional model for the origin of monocots. Am J Bot.

[19] Koren S, Rhie A, Walenz BP, Dilthey AT, Bickhart DM, Kingan SB (2018). De novo assembly of haplotype-resolved genomes with trio binning. Nat Biotechnol.

[20] Pellicer J, Leitch IJ (2020). The plant DNA C-values database (release 7.1): an updated online repository of plant genome size data for comparative studies. New Phytol..

[21] Jain M, Koren S, Miga KH, Quick J, Rand AC, Sasani TA (2018). Nanopore sequencing and assembly of a human genome with ultra-long reads. Nat Biotechnol.

[22] Charalampous T, Kay GL, Richardson H, Aydin A, Baldan R, Jeanes C (2019). Nanopore metagenomics enables rapid clinical diagnosis of bacterial lower respiratory infection. Nat Biotechnol.

[23] De Coster W, De Rijk P, De Roeck A, De Pooter T, D’Hert S, Strazisar M (2019). Structural variants identified by Oxford Nanopore PromethION sequencing of the human genome. Genome Res.

[24] Wang M, Fu A, Hu B, Tong Y, Liu R, Liu Z (2021). Nanopore targeted sequencing for the accurate and comprehensive detection of SARS-CoV-2 and other respiratory viruses. Small.

[25] Lang D, Zhang S, Ren P, Liang F, Sun Z, Meng G (2020). Comparison of the two up-to-date sequencing technologies for genome assembly: HiFi reads of Pacific Biosciences Sequel II system and ultralong reads of Oxford Nanopore. GigaScience..

[26] Shang L, Li X, He H, Yuan Q, Song Y, Wei Z (2022). A super pan-genomic landscape of rice. Cell Res.

[27] Closing the gap in plant genomes. Oxford Nanopore Technologies. 2022. https://nanoporetech.com/resource-centre/closing-gap-plant-genomes. Accessed 27 July 2022.

[28] Edge P, Bansal V (2019). Longshot enables accurate variant calling in diploid genomes from single-molecule long read sequencing. Nat Commun.

[29] Medaka. Oxford Nanopore Technologies. 2022. https://github.com/nanoporetech/medaka. Accessed 25 Mar 2022.

[30] Ahsan MU, Liu Q, Fang L, Wang K (2021). NanoCaller for accurate detection of SNPs and indels in difficult-to-map regions from long-read sequencing by haplotype-aware deep neural networks. Genome Biol.

[31] Shafin K. kishwarshafin/pepper. 2022. https://github.com/kishwarshafin/pepper. Accessed 25 Mar 2022.

[32] Jones A, Torkel C, Stanley D, Nasim J, Borevitz J, Schwessinger B (2021). High-molecular weight DNA extraction, clean-up and size selection for long-read sequencing. PLOS ONE..

[33] Cheng H, Concepcion GT, Feng X, Zhang H, Li H (2021). Haplotype-resolved de novo assembly using phased assembly graphs with hifiasm. Nat Methods.

[34] Bonito. Oxford Nanopore Technologies. 2022. https://github.com/nanoporetech/bonito. Accessed 27 Mar 2022.

[35] De Coster W, D’Hert S, Schultz DT, Cruts M, Van Broeckhoven C (2018). NanoPack: visualizing and processing long-read sequencing data. Bioinformatics..

[36] Li H (2018). Minimap2: pairwise alignment for nucleotide sequences. Bioinformatics..

[37] Pomoxis—bioinformatics tools for nanopore research. Oxford Nanopore Technologies. 2022. https://github.com/nanoporetech/pomoxis. Accessed 27 Mar 2022.

