# Supplementary material for: Species-specific basecallers improve actual accuracy of nanopore sequencing in plants
Source: Plant Methods. 2022 Dec 14;18:137. doi: 10.1186/s13007-022-00971-2 (PMC9749173; doi:10.1186/s13007-022-00971-2)
Supplement: Supplementary file 1 — Additional file 1. Additional results, additional tables S1–S4 and additional figures S1–S9. [file 13007_2022_971_MOESM1_ESM.docx]

# Additional file 1

# Additional Results

## Duplex reads

After basecalling the Guppy-6-R10 library consisted of 32.85 Gbp of sequence in 2,376,108 reads of which 1.77 Gbp (5.38%) of sequence in 133,969 reads (5.64%) were identified as duplex reads. Alignment of Guppy-duplex reads to the pseudo-haploid HiFi genome was highly unsuccessful, only 0.16% of duplex reads aligned. To determine if template and complement reads were correctly paired and combined into duplex reads we aligned all duplex read pairs and calculated percent similarity. Read pairs were found to be highly similar (81.9% had +90% sequence similarity), indicating that read pairs were likely to have been correctly identified. Additionally, we attempted to align duplex reads to the HiFi genome with an alternative long-read aligner, NGMLR [1]. NGMLR achieved a similarly low alignment rate. As too few duplex reads aligned to the HiFi genome we can not assess duplex read accuracy.

| **Quality metric** | **Basecaller model** | **Read quality scores** | **Read Identity** | **Read N50 (Kbp)** | **Average read length (Kbp)** |
| --- | --- | --- | --- | --- | --- |
| Percent | Guppy-duplex | 89.81% ± 0.57% | 95.32% ± 5.29% | 18.03 | 13.19 ± 9.07 |
|  | Simplex-of-duplex | 87.13% ± 0.84% | 97.21% ± 3.23% | 23.81 | 17.39 ± 12.81 |
| Phred score | Guppy-duplex | 9.93 ± 0.26 | 27.99 ± 22.02 | --- | --- |
|  | Simplex of duplex | 8.91 ± 0.28 | 17.81 ± 4.94 | --- | --- |

**R10.4 read library quality statistics.** Average read quality scores and read identity for Duplex *P. stellatum* R10.4 read library and the simplex reads that were collapsed into duplex reads. Average read quality scores were calculated per read, and the overall average calculated. Read identity is calculated per read against the HiFi genome and averaged. Note: only 212 duplex reads aligned to *P. stellatum’s* HiFi genome, statistics for Guppy-duplex and Simplex-of-duplex should be viewed with caution. Averages are shown with standard deviations.

## R10.4 read pair analysis

Produced by the duplex basecalling pipeline is a read pair text file, which lists read pair names (template and complement strands). Read pairs were extracted from the library fastq file and percent of aligned bases calculated with MUMmer [2]. Statistics (average, standard deviation, and percent above 90%) were calculated within bash.

## References

1. Sedlazeck FJ, Rescheneder P, Smolka M, Fang H, Nattestad M, von Haeseler A, et al. Accurate detection of complex structural variations using single-molecule sequencing. Nat Methods. 2018 Jun;15(6):461–8.

2. Marçais G, Delcher AL, Phillippy AM, Coston R, Salzberg SL, Zimin A. MUMmer4: A fast and versatile genome alignment system. Darling AE, editor. PLOS Comput Biol. 2018 Jan 26;14(1):e1005944.

|  | ***X. johnsonii*** | | ***P. stellatum*** | |
| --- | --- | --- | --- | --- |
|  | Raw reads | Assembly reads | Raw reads | Assembly reads |
| **n50 (Kbp)** | 19.79 | 19.61 | 19.98 | 19.81 |
| **number** | 2,210,350 | 1,838,893 | 1,131,096 | 956178 |
| **longest (Kbp)** | 53.79 | 49.37 | 53.04 | 47.17 |
| **Mean read quality** | 29.8 | 31.4 | 29.4 | 30.9 |
| **Total (Mbp)** | 44,056 | 36,346 | 22,703 | 19,045 |
| **Diploid coverage** | 15.3 | 12.6 | 18.2 | 15.3 |

**Table S1. HiFi read library statistics**. Statistics for all read libraries before and after filtering.

|  | ***P. stellatum* R9.4.1** | | ***P. stellatum* R10.4** | | ***X. johnsonii* R9.4.1** | |
| --- | --- | --- | --- | --- | --- | --- |
|  | **Basecalling** | **Training** | **Basecalling** | **Training** | **Basecalling** | **Training** |
| **Training subset 1** | 34:02 | 14:38 | 31:36 | 26:27 | 41:57 | 25:48 |
| **Training subset 2** | 36:13 | 15:10 | 29:56 | 41:58 | 41:13 | 22:55 |
| **Training subset 3** | 36:55 | 14:47 | 26:17 | 42:16 | --- | --- |
| **Total** | 107:10 | 44:35 | 87:49 | 110:41 | 83:10 | 48:43 |
| **Grand total** |  | 151:45 |  | 198:30 |  | 131:53 |

**Table S2. Compute run times for basecaller training jobs**, presented in hours and minutes (h:min). Both basecalling and training were performed by ONT Bonito. All jobs were run with 12 Intel Xeon Platinum 8268 (Cascade Lake) 2.9 GHz CPU cores and a single Nvidia Tesla Volta V100-SXM2-32GB GPU. The maximum system memory used was: basecalling = 367 GB; training = 65 GB.


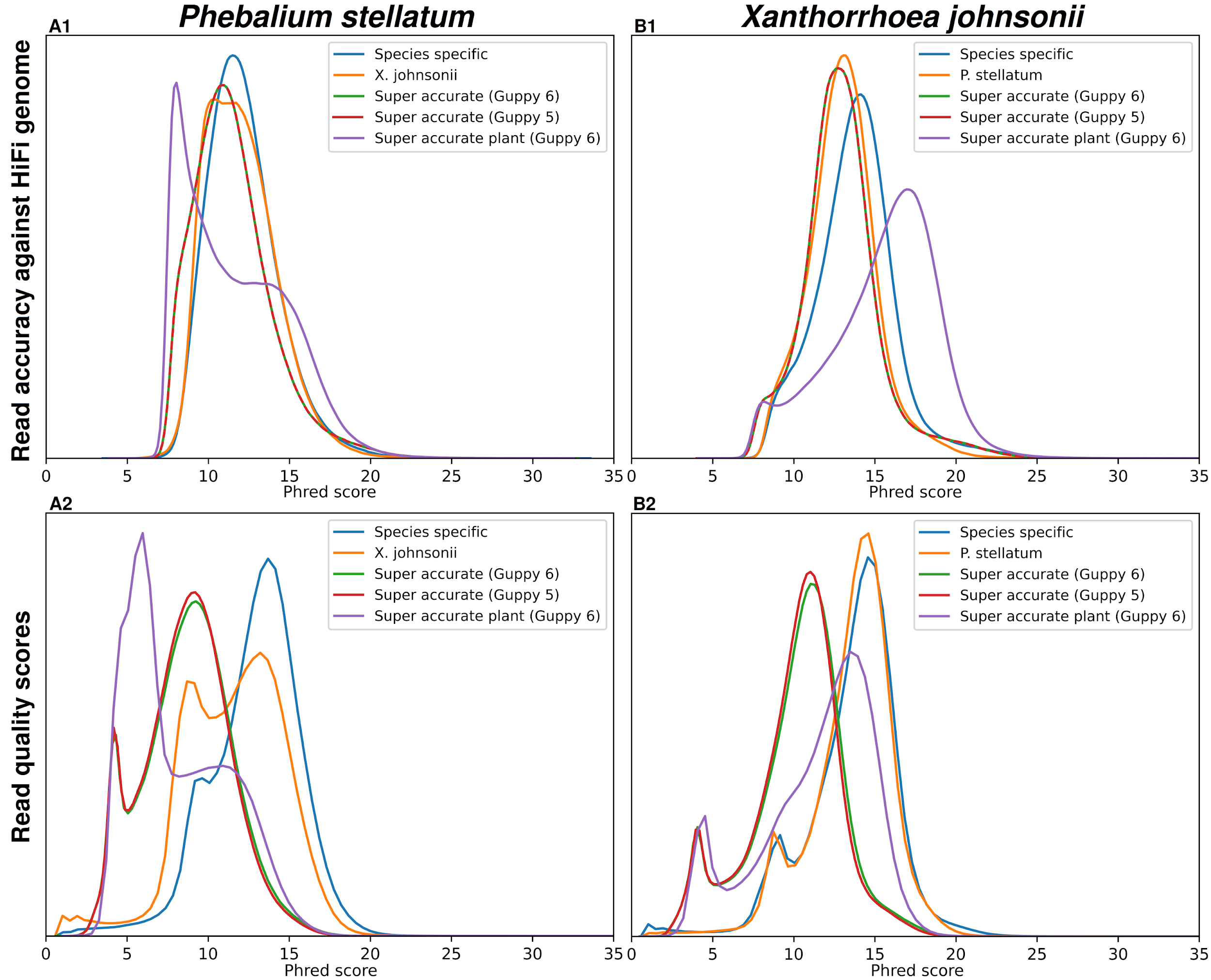


**Figure S1.** **Distribution of R9.4.1 read library quality statistics, in Phred scores.** A1 and A2 contain statistics for *P. stellatum*; B1 and B2 contain statistics for *X. johnsonii*. A1) Distribution of read identities (compared to the pseudo-haploid HiFi genome (truth set)) for each of *P. stellatum’s* read libraries. A2) Distribution of average read quality scores (obtained from basecalled fastq files) for all *P. stellatum’s* reads libraries. B1) Distribution of read identities (compared to the pseudo-haploid HiFi genome (truth set)) for each of *X. johnsonii’s* read libraries. B2) Distribution of average read quality scores (obtained from basecalled fastq files) for all *X. johnsonii’s* reads libraries.

|  | ***P. stellatum*** | | ***X. johnsonii*** | |
| --- | --- | --- | --- | --- |
| **Basecaller model** | **Read quality scores** | **Read Identity** | **Read quality scores** | **Read Identity** |
| Guppy 5  super accurate | 8.65 ± 2.67 | 10.95 ± 0.17 | 10.07 ± 2.85 | 12.33 ± 0.14 |
| Guppy 6  super accurate | 8.75 ± 2.74 | 10.95 ± 0.17 | 10.21 ± 2.90 | 12.33 ± 0.14 |
| Guppy 6  super accurate - plant | 8.16 ± 3.10 | 10.69 ± 0.16 | 11.44 ± 3.43 | 14.18 ± 0.16 |
| Reciprocal species-specific | 11.55 ± 3.08 | 11.54 ± 0.14 | 13.51 ± 2.60 | 12.54 ± 0.12 |
| Species-specific | 12.86 ± 2.79 | 11.70 ± 0.13 | 13.56 ± 2.88 | 13.16 ± 0.12 |

**Table S3. Basecaller quality.** Average read quality scores (quality scores) for each dataset were calculated, averaged and reported as Phred scores. Read identities show the average read similarity to the truth dataset and are also reported as Phred scores. Averages are shown with standard deviations.


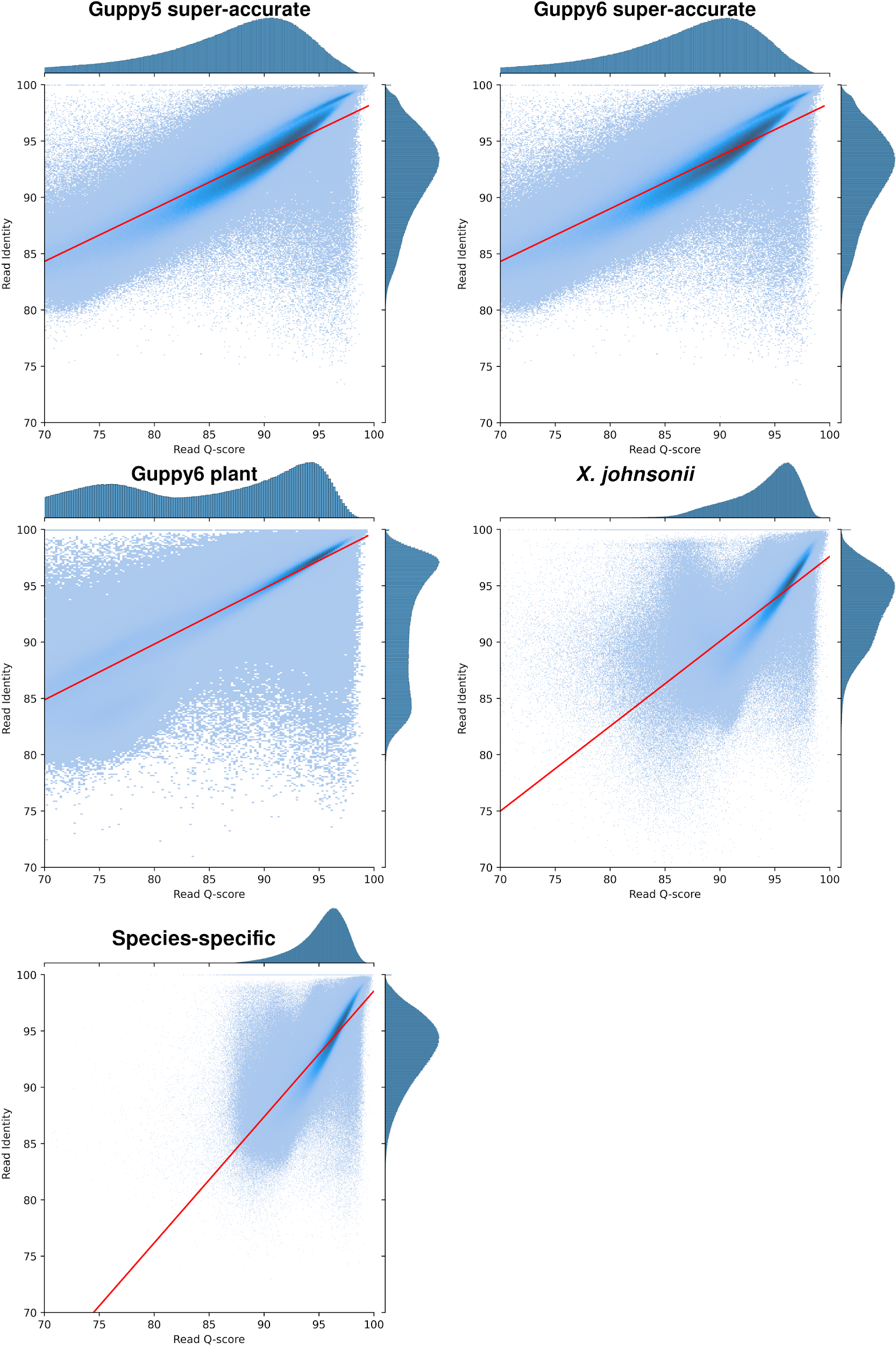


**Figure S2.** Scatter plot with linear regression of all *P. stellatum* read datasets, in percentages. X-axis shows read average quality scores in percentages, while the y-axis shows the read identity (accuracy against the HiFi truth genome).


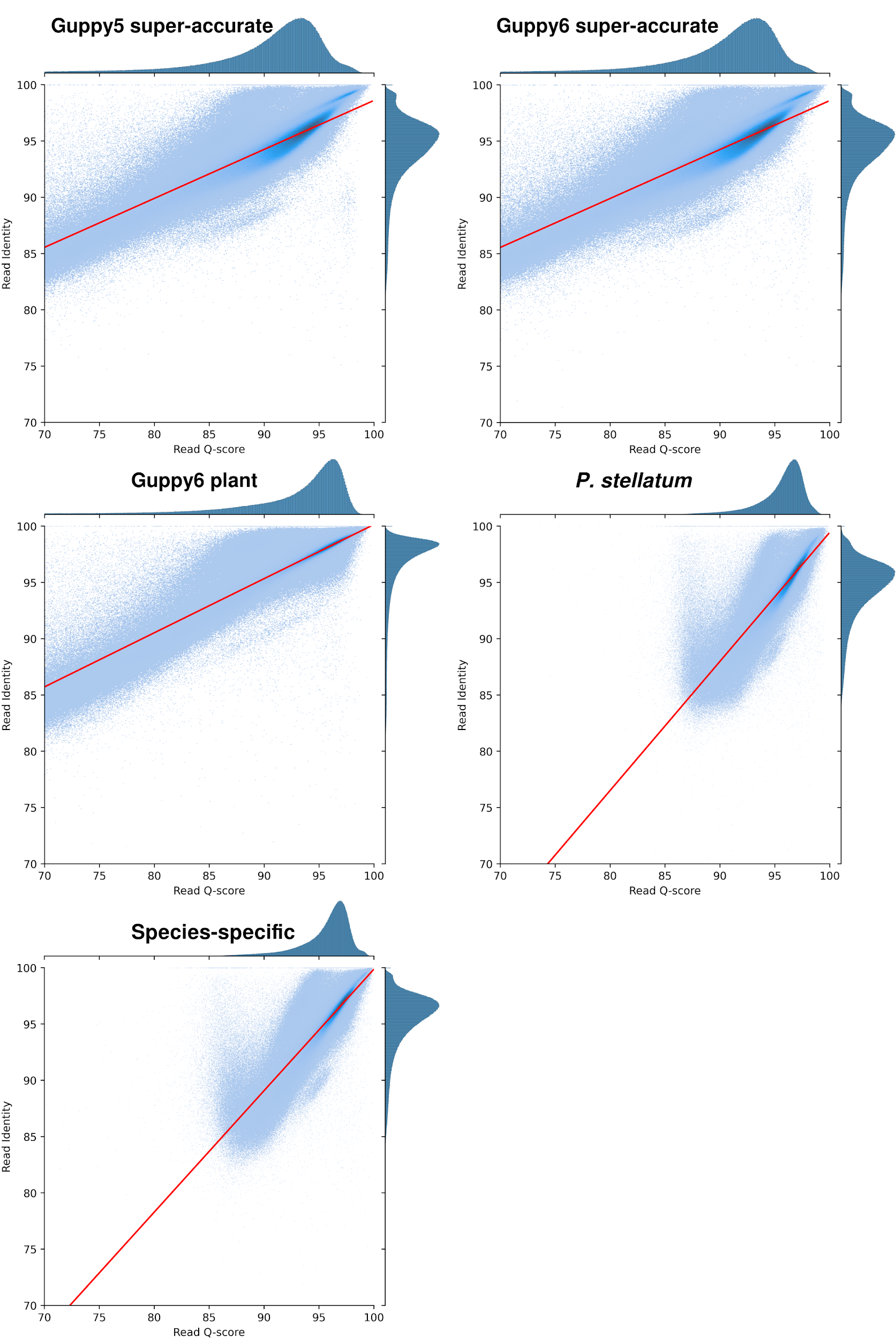


**Figure S3.** Scatter plot with linear regression of all *X. johnsonii* read datasets, in percentages. X-axis shows read average quality scores, while the y-axis shows the read identity (accuracy against the HiFi truth genome.


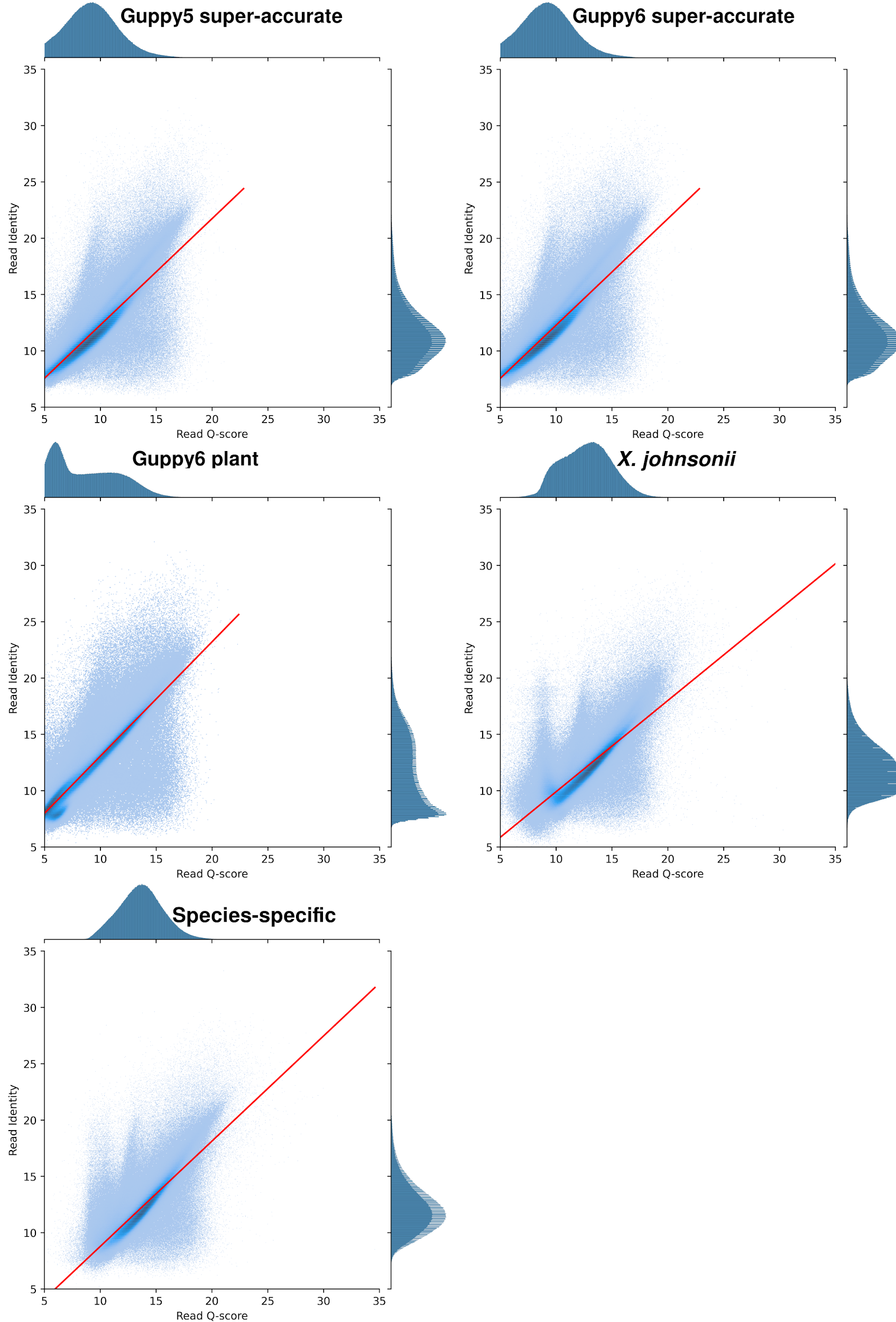


**Figure S4.** Scatter plot with linear regression of all *P. stellatum* read datasets, in Phred scores. X-axis shows read average quality scores, while the y-axis shows the read identity (accuracy against the HiFi truth genome).


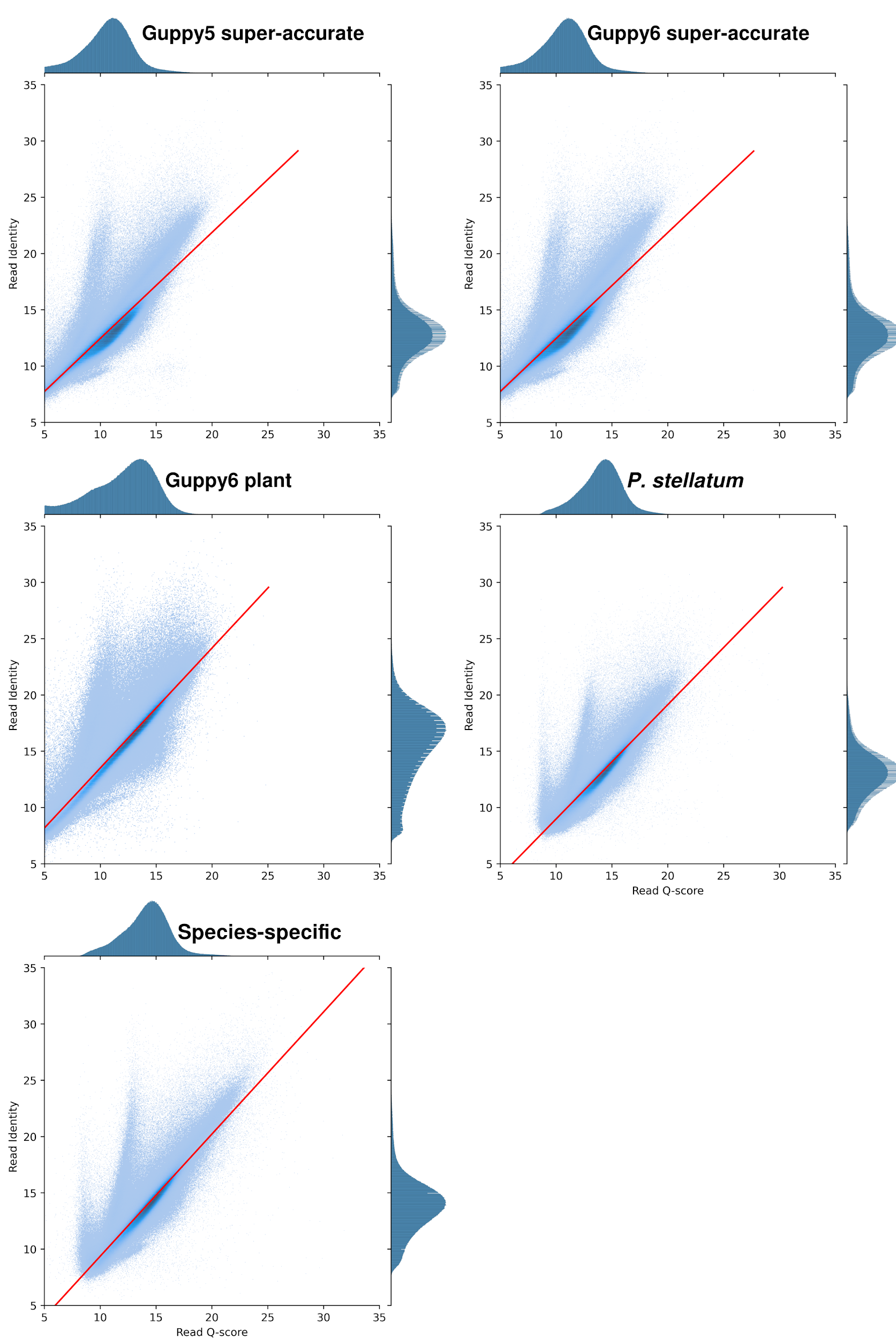


**Figure S5.** Scatter plot with linear regression of all *X. johnsonii* read datasets, in Phred scores. X-axis shows read average quality scores, while the y-axis shows the read identity (accuracy against the HiFi truth genome).


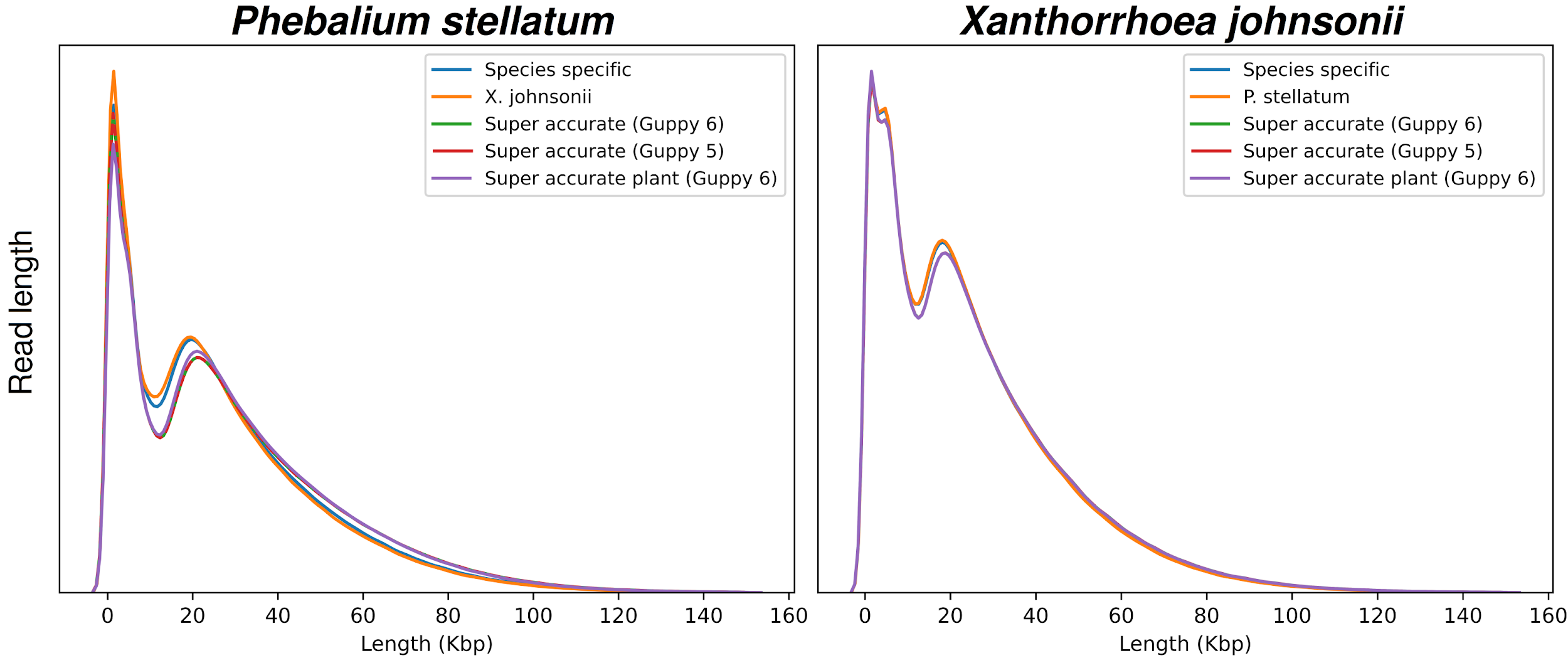
 **Figure S6.** Read length distribution for all R9.4.1 basecalled read datasets.


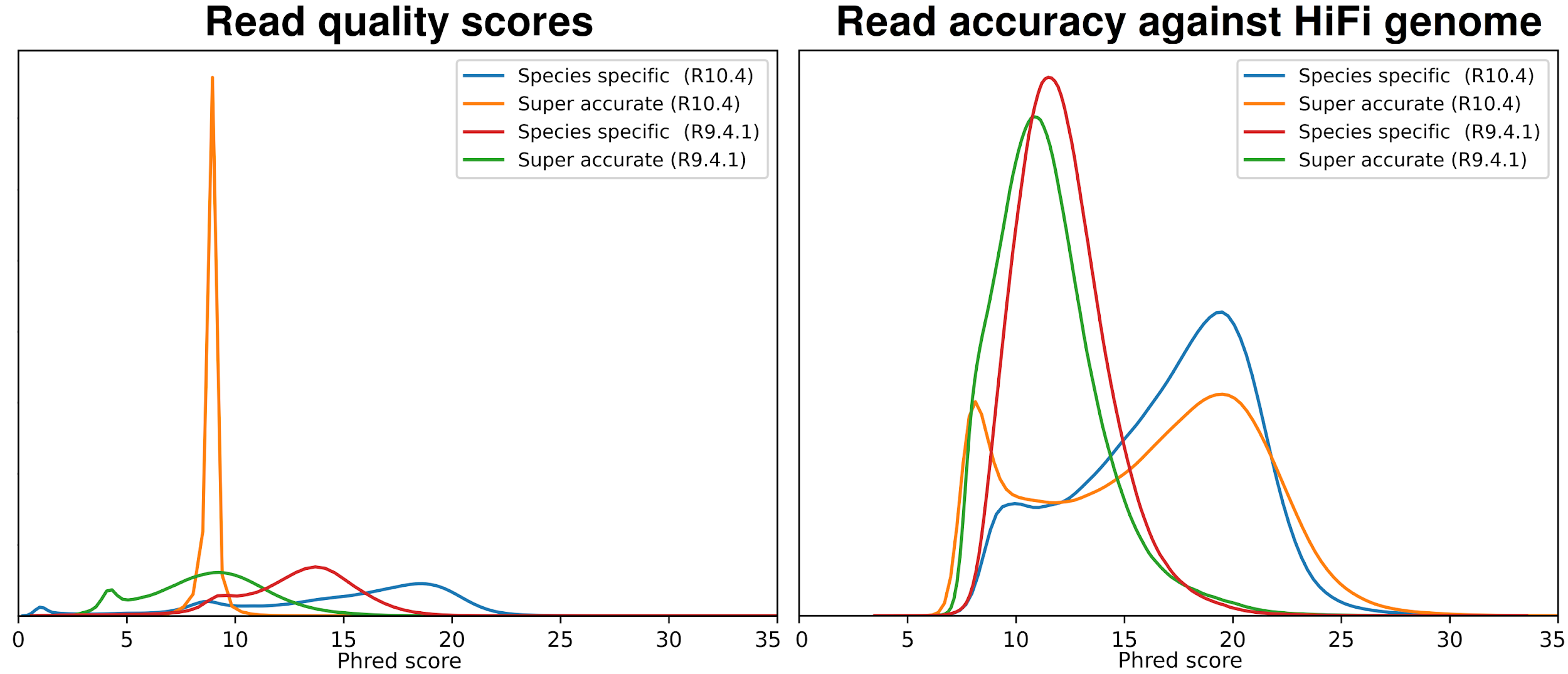


**Figure S7.** **Distribution of R10.4 read library quality statistics, in Phred scores.** Left: Distribution of average read quality scores (obtained from basecalled fastq files) for all *P. stellatum’s* reads libraries. Right: Distribution of read identities (compared to the pseudo-haploid HiFi genome (truth set)) for each of *P. stellatum’s* read libraries.

| **Basecaller model** | **Read quality scores** | **Read Identity** |
| --- | --- | --- |
| Guppy-6-R10.4 | 8.88 ± 0.62 | 6.64 ± 6.73 |
| Species-specific R10.4 | 12.86 ± 2.79 | 11.70 ± 0.13 |

**Table S4. R10.4 read library quality statistics, in Phred scores.** Average read quality scores (quality scores) for each dataset were calculated, averaged and reported as percent accuracy. Read identities show the average read similarity to the truth dataset and are also reported as percent accuracy. Averages are shown with standard deviations.


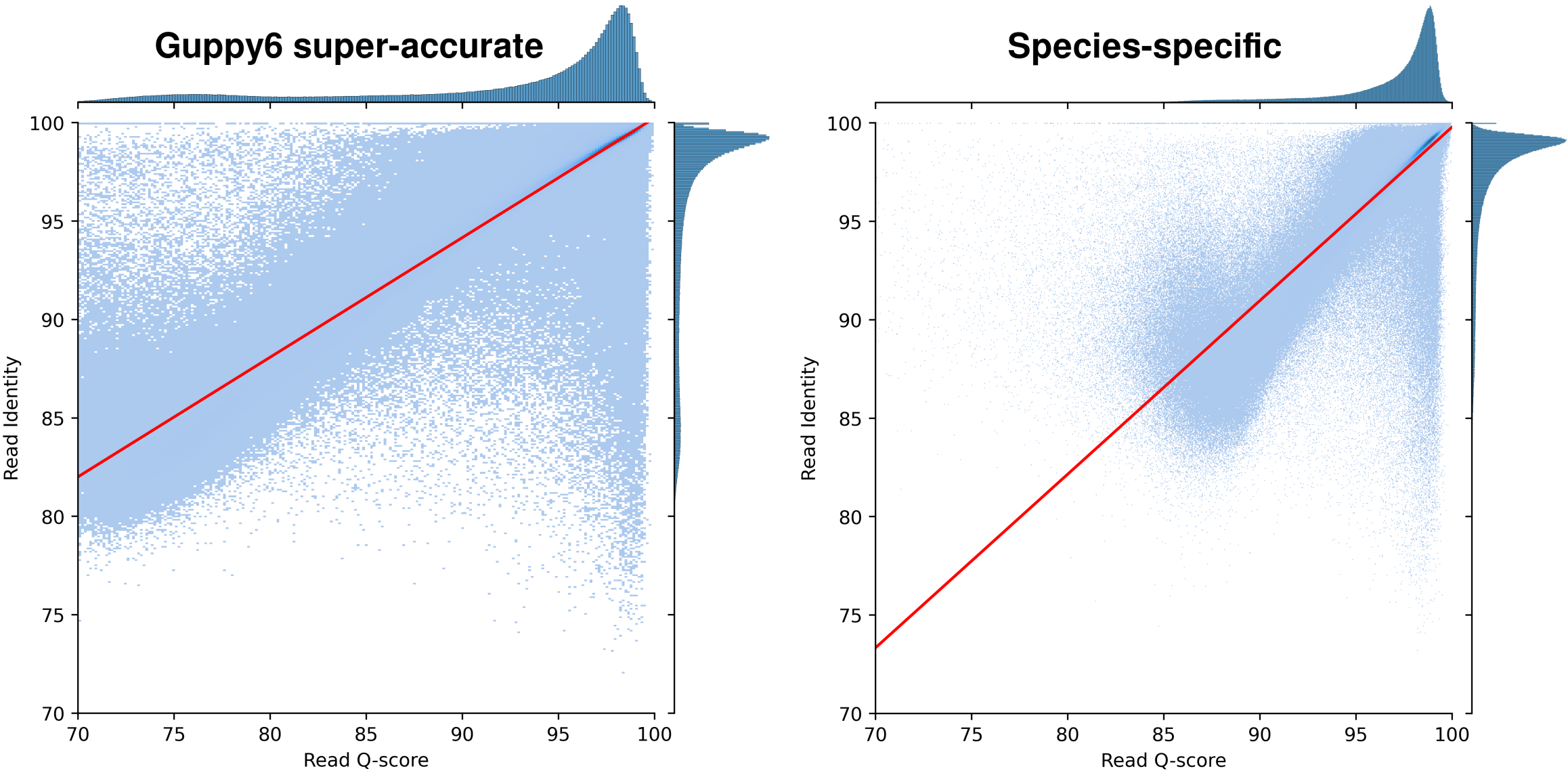


**Figure S8.** Scatter plot with linear regression of all *X. johnsonii* read libraries, in percentages. X-axis shows read average quality scores, while the y-axis shows the read identity (accuracy against the HiFi truth genome).


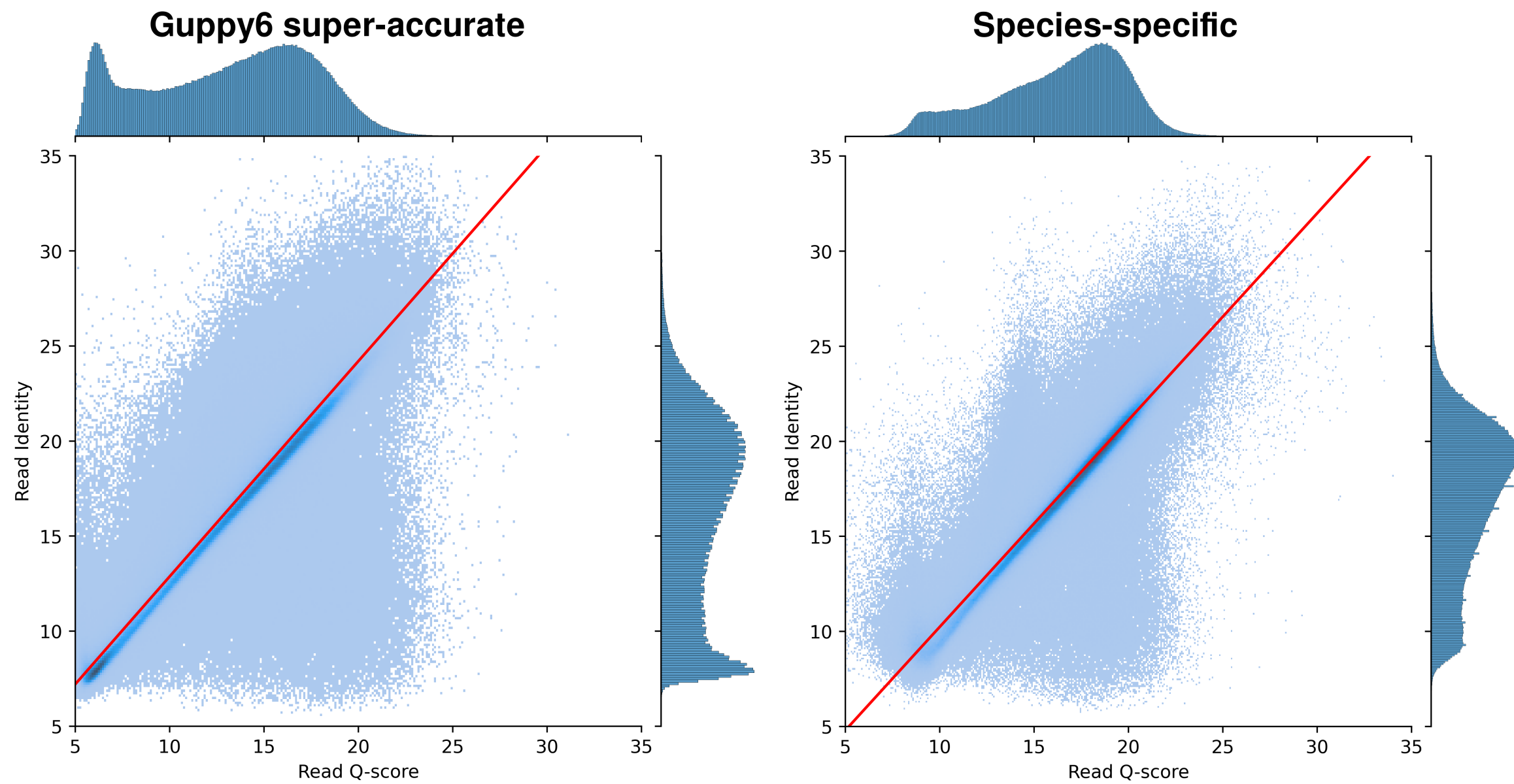


**Figure S9.** Scatter plot with linear regression of all *X. johnsonii* read libraries, in Phred scores. X-axis shows read average quality scores, while the y-axis shows the read identity (accuracy against the HiFi truth genome).
